# Etymologia: *Angiostrongylus*

**DOI:** 10.3201/eid2406.ET2406

**Published:** 2018-06

**Authors:** Ronnie Henry

**Keywords:** Angiostrongylus, Angiostrongylus cantonensis, Angiostrongylus costaricensis, Parastrongylus cantonensis, parasites, rat lungworm, roundworms, eosinophilic meningitis, China

## *Angiostrongylus* [anʺje-o-stronʹjĭ-ləs]

From the Greek *angeion* (“vessel”) + *strongylos* (“round”), *Angiostrongylus* (Figure) is a genus of parasitic nematodes (roundworms) in the family *Angiostrongylidae*, 2 species of which are known to parasitize humans. *A. cantonensis* (commonly known as rat lungworm) was first described in 1935 (as *Pulmonema cantonensis*) from rats in Canton, China. It is the most common cause of eosinophilic meningitis in Asia and the Pacific Basin, but cases have been reported in many parts of the world. *A. costaricensis* roundworms were first described in 1971 in Costa Rica from surgical specimens from children with eosinophilic infiltration in the abdominal cavity. The distribution of this species ranges from the southern United States to northern Argentina.

**Figure:**
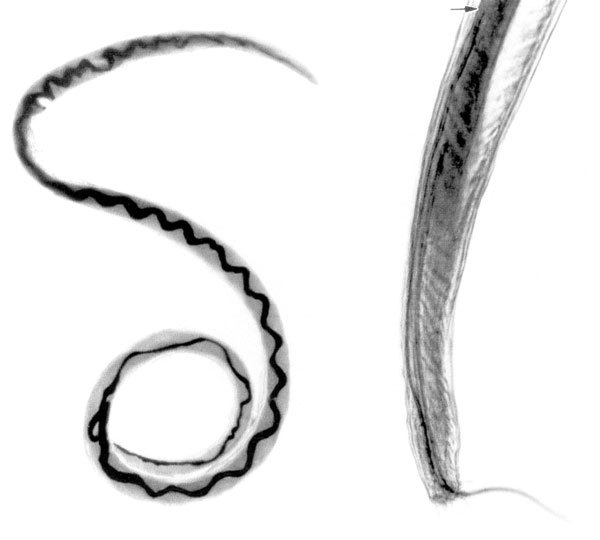
Adult *Angiostrongylus cantonensis *nematode recovered from rat lung. Image from Enzootic *Angiostrongylus cantonensis *in Rats and Snails after an Outbreak of Human Eosinophilic Meningitis, Jamaica, John F. Lindo et al, Emerging Infectious Diseases, Vol. 8, No. 3, March 2002.

There is still debate about what taxonomic name should be used. *A. cantonensis* remains in general use, but some researchers suggest it should be changed to *Parastrongylus cantonensis* on the basis of the morphology of the adult male bursa and the definitive host being rats.

## References

[1] Chen HT. A new pulmonary nematode, *Pulmonema cantonensis*, n.g., n.sp. in Canton rats [in French]. Ann Parasitol. 1935;13:312–7. 10.1051/parasite/1935134312

[2] Cowie RH. Biology, systematics, life cycle, and distribution of *Angiostrongylus cantonensis*, the cause of rat lungworm disease. Hawaii J Med Public Health. 2013;72(Suppl 2):6–9. 23901372 PMC3689493

[3] Morera P, Céspedes R. *Angiostrongylus costaricensis* n. sp. (Nematoda: Metastrongyloidea), a new lungworm occurring in man in Costa Rica. Rev Biol Trop. 1970;18:173–85. 5527668

[4] Simner PJ. Medical parasitology taxonomy update: January 2012 to December 2015. J Clin Microbiol. 2016;55:43–7. 10.1128/JCM.01020-16 27440818 PMC5228259

[5] Ubelaker JE. Systematics of species referred to the genus *Angiostrongylus.* J Parasitol. 1986;72:237–44. 10.2307/3281599 3525794

